# A compilation of ab-initio calculations of embrittling potencies in binary metallic alloys

**DOI:** 10.1016/j.dib.2015.11.024

**Published:** 2015-12-01

**Authors:** Michael A. Gibson, Christopher A. Schuh

**Affiliations:** Department of Materials Science and Engineering, Massachusetts Institute of Technology, 77 Massachusetts Avenue, Cambridge, MA, USA

## Abstract

Segregation-induced changes in interfacial cohesion often control the mechanical properties of metals. The change in the work of separation of an interface upon segregation of a solute to the interface, termed the embrittling potency, is an atomic-level quantity used to predict and understand embrittlement phenomena. We present a compilation of calculations of embrittling potencies, along with references for these calculations. A discussion of this data is made in a separate article (Gibson and Schuh, 2016 [1]).

**Specifications Table**

**Table:** 

Subject area	Materials Science.
More specific subject area	Metallurgy.
Type of data	Tables.
How data was acquired	Literature Review.
Data format	Raw.
Experimental factors	NA.
Experimental features	NA.
Data source location	
Data accessibility	Data is with the article.

**Value of the data**

• This compilation of materials data may be analyzed to provide insight in atomic mechanisms of embrittlement across systems.
• The values may serve as a quick, comprehensive reference to check which solutes may embrittle or increase boundary cohesion in a given solvent, aiding alloy design and failure analysis.
• Comparison across different boundary types may give insight into the degree of anisotropy of embrittling potencies.
• This database may facilitate comparison to previous studies in the future, and demonstrate which systems or boundaries merit further study, and which systems have yet to be studied.

## 1 Data

This data set is an aggregation of the embrittling potencies in binary metallic alloys from a large number of previously published studies. As such, the relevant methods for obtaining each individual data point are those from the original studies, and are listed in the data. The value of the present data is simply in aggregating all of these results in a single location such that it is computable and searchable. As such, the relevant methods for this data are the methods used in reviewing the literature. The data is available in computable form as a pair of .csv files, as well as two human-readable tables, included in the present data article. explicitly discussed in a separate article by the authors [1].

## 2 Experimental design, materials and methods

### 2.1 The data gathering process

An attempt was made to gather all calculations of embrittling potencies in the literature. In doing so, several methods were used to gather studies:

1. All studies cited by Ref. [2] were reviewed.
2. Citation alerts on Google Scholar for the keywords: “Grain boundary Segregation”, “surface segregation”, and “GB embrittlement” ⋁ “Grain boundary embrittlement” have been in place since early 2014 to capture recent publications on the subject.
3. A backward search for previous publications on embrittling potencies was conducted by searching through all of the references contained in each publication we found to see if any other calculations of embrittling potencies were made.
4. A forward search was conducted by reviewing the citing articles for all of the highest-impact works on grain boundary embrittlement.
5. Google Scholar searches for the term “embrittling potency”.

We found that performing a backward search from the publications captured by our Google Scholar alerts published during 2015 yielded almost exclusively studies that we had already recorded, lending confidence to our belief that we have captured a representative, if not exhaustive, list of studies. All calculations were systematically recorded. For the reader׳s reference, any DFT study that calculated a grain boundary segregation energy or simply conducted a qualitative investigation of the charge distribution at the grain boundary is also included in the below tables, although such studies did not contribute to the quantitative study of the embrittling potencies which was the purpose of this data article.

The present authors felt it was best to separate out data that might be questionable for use in future, quantitative analyses of grain boundary cohesion from the data that might safely be included in such studies. The criteria for inclusion of the data in these two data sets is laid out below:

• If multiple studies examined the same solute at the same GB via the same methods, and one study reproduced the results of a previous study in terms of segregation energies and embrittling potencies, and the later study then showed that a more stable site for the solute exists at the GB, then only the data point pertaining to the more stable site was retained in the quantitative data set. In cases where authors disagreed, both studies were retained in an effort for impartial review.
• If the same research group conducted multiple, essentially analogous calculations (i.e. the same solute at the same GB in the same solvent), the most recent calculation was used in the quantitative data set. In our experience, though, these calculations tended to be close to one another, so the choice of study is unlikely to have a large impact on the analysis.
• If a DFT cluster calculation was performed, and there exists a more accurate, periodic boundary condition calculation of the same solute in the same solvent, the cluster calculation was excluded from the quantitative data set.
• Calculations from Finnis–Sinclair potentials (which differ substantially from the rest of the data) were not included in the analysis. This is consistent with Ref. [2]; the present authors are not the first to make this exclusion.
• Calculations from publications which contained insufficient detail for the work to be reproducible or made unphysical assumptions were not included.

The data includes four embedded atom method (EAM) calculations, and eight estimates from the experimentally measured difference in free energies for segregation (discussed below), with the remainder of calculations coming from density functional theory (DFT) calculations. Any calculations for which an embrittling potency is not listed are systems wherein grain boundary segregation has been studied from a theoretical perspective, but an embrittling potency was not specifically calculated and could not be derived from the data presented. These pairs and references are nonetheless listed so that the interested reader may more easily find studies on grain boundary segregation for specific systems.

The experimental values of the embrittling potencies are computed from the thermodynamic theory of Hirth, Rice, and Wang [3], [4], [5]. In a simplified interpretation, and in the dilute limit, the embrittling potency of a segregant is equal to the difference in the grain boundary and surface segregation energies:

(1) $$∆E_{B}=W_{\mathrm{pure}}^{\mathrm{sep}}-W_{\mathrm{impure}}^{\mathrm{sep}}=∆E_{\mathrm{gb}}^{\mathrm{seg}}-∆E_{\mathrm{surf}}^{\mathrm{seg}}$$

The difference in the internal energies of segregation is approximately equal to the difference between the experimentally measurable free energies of segregation, to the surface and the GB, $∆G_{\mathrm{GB}}^{\mathrm{seg}}$ and $∆G_{\mathrm{surf}}^{\mathrm{seg}}$.^1^ However, while the analysis for the DFT studies was made assuming that the solute remained in the same site during fracture, such a constraint is not possible during the experimental measurements of segregation behavior. Thermodynamically, the imposition of a constraint guarantees that the work needed to perform a process is larger than in the absence of the constraint. Thus, the experimentally measured embrittling potencies should be considered an upper bound when compared with the theoretically computed embrittling potencies. Despite this difference, previous authors have shown that the embrittling potencies computed from a theoretical and experimental perspective are in fair agreement [2], [6].

Table S1 is a table of embrittling potencies suitable for quantitative analysis. Acronyms used in Table S1 represent:

**DFT**: Density Functional Theory
**PBC**: Periodic Boundary Conditions – used as shorthand for calculations in which the grain boundary was constructed such that two grain orientations are tessellated periodically next to one another.
**Slab**: Used as shorthand for calculations in which two adjacent grains create a GB, which is then surrounded by vacuum in the z direction. This is in contrast to the above PBC calculations.
**XC Functional**: Exchange-Correlation functional
**MD**: Molecular Dynamics
**EAM**: Embedded Atom Method
**LDA**: Local Density Approximation
**GGA**: Generalized Gradient Approximation
**FLAPW**: Full potential Linearized Augmented Plane Wave
**PW91**: A flavor of GGA
**PBE**: A flavor of GGA
**LMTO**: Linear Muffin Tin Orbitals
**FP**: Full Potential
**LCAO**: Linear Combination of Atomic Orbitals
**DMol**: Refers to software by Accelrys used for cluster calculations in DFT

In similar spirit to the inclusion of studies in Table 1 where an embrittling potency was not calculated, Table 2 lists calculations and their associated references that are deemed not as suitable for quantitative analysis. These are nonetheless listed so that the interested reader may more easily find studies on grain boundary segregation and embrittlement for specific systems.

## Footnotes

1 This approximation is true if the entropy due to a solute atom occupying the surface site is approximately equal to that for a solute atom occupying a grain boundary site. This difference is likely non-zero, but the T∆S term is likely negligible relative to the energetic differences at the surface and the GB.
