# Supplementary material for: A compilation of ab-initio calculations of embrittling potencies in binary metallic alloys
Source: Data Brief. 2015 Dec 1;6:143–8. doi: 10.1016/j.dib.2015.11.024 (PMC4706572; doi:10.1016/j.dib.2015.11.024)
Supplement: Supplementary file 4 — Supplementary material [file mmc4.pdf]

Table 1: Embrittling potencies for use in quantitative analysis.

Base Metal	Solute	GB	Method	XC Functional, if DFT	ΔE_B(kJ/mol) (Embrittling potency)	Reference	Notes
Al	Ca	Sigma 9 (2-21)/[110]	DFT, PBC's	LDA	19	[7]	
Al	Ca	Sigma 11(113)[110]	DFT - PBC's	GGA (PW91)	77	[8]	
Al	Cr	Sigma 5 (012)[100]	DFT, slab	GGA	-155.6	[9]	
Al	Mg	Sigma 5 (012)[100]	DFT FLAPW, slab	GGA	-11	[10]	
Al	Mg	Sigma 5 (012)[100]	DFT, slab	GGA	-13	[9]	
Al	Mg	Sigma 11 (111)	DFT, PBC's	GGA (PW91)	12	[11]	
Al	Mg	Sigma 11(113)[110]	DFT - PBC's	GGA (PW91)	13	[8]	
Al	Na	Sigma 9 (2-21)/[110]	DFT, PBC's	LDA	145	[7]	
Al	Na	Sigma 5 (012)[100]	DFT FLAPW, slab	GGA	60	[12]	
Al	Na	Sigma 5 (012)[100]	DFT FLAPW, slab	GGA	NA	[13]	
Al	Na	Sigma 11(113)[110]	DFT - PBC's	GGA (PW91)	155	[8]	
Al	Ni	Sigma 5 (012)[100]	DFT, slab	GGA	-94	[9]	
Al	B	Sigma 9 (2-21)/[110]	DFT FLAPW, slab	GGA	-18	[14]	
Al	B	Sigma 5 (210)	DFT, slab	GGA	-15	[9]	
Al	Zn	Sigma 5 (210)	DFT FLAPW, slab	GGA	4.8	[15]	
Al	Zr	Sigma 5 (210)	DFT, slab	GGA	-158	[9]	
Al	P	Sigma 5 (210)	DFT, slab	GGA	36.6	[9]	
Al	Si	Sigma 5 (210)	DFT, slab	GGA	6.7	[9]	
Al	K	Sigma 11(113)[110]	DFT - PBC's	GGA (PW91)	268	[8]	
Al	Sr	Sigma 11(113)[110]	DFT - PBC's	GGA (PW91)	164	[8]	
Cr	Fe	Sigma 3 {111}	DFT - PBC's	GGA	-6.1	[16]	
Cr	Fe	Sigma 5 (012)[100]	DFT - PBC's	GGA	-1.9	[16]	
Cu	Ag	Sigma 5 (310)[001]	DFT, PBC's	LDA	-1.9	[17]	
Cu	Bi	37 degree {001} tilt	DFT	LDA		[18]	
Cu	Bi	Sigma 19a GB	DFT - PBC'S	LDA	56.4	[19]	
Cu	Bi	Sigma 5 (310)[001]	DFT, PBC's	LDA	142.6	[17]	
Cu	Na	Sigma 5 (310)[001]	DFT, PBC's	LDA	160	[17]	
Cu	Pb	Sigma 19a GB	DFT- PBC'S	LDA	48.7	[19]	

Table 1 (continued): Embrittling potencies for use in quantitative analysis.

Base Metal	Solute	GB	Method	XC Functional, if DFT	ΔE_B(kJ/mol) (Embrittling potency)	Reference	Notes
Cu	B	Sigma 5 (310)[001]	DFT - PBC's	FP LMTO with LDA (Von Barth and Hedin)	-54	[20]	
Cu	S	Sigma 5 (001)	DFT	GGA - PBE	91	[21]	
Cu	Cl	Sigma 5 (001)	DFT	GGA - PBE	251.6	[21]	
Cu	S	Sigma 7 (111)	DFT	GGA - PBE	73.6	[21]	
Cu	Cl	Sigma 7 (111)	DFT	GGA - PBE	218.5	[21]	
Fe	Al	{111}	DFT - PBC's	GGA	-0.96	[22]	
Fe	Co	Sigma 3 [1-10](111)	DFT (Dmol cluster)	GGA	24	[23]	
Fe	Co	Sigma 3 {111}	DFT FLAPW – slab	GGA	4.8	[24]	
Fe	Cr	Sigma 3 [1-10](111)	DFT (Dmol cluster)	GGA	-41.4	[23]	
Fe	Cr	Sigma 5 [001](310)	MD - EAM	N/A	32.8	[25]	
Fe	Cu	Sigma 3 [1-10](111)	DFT - PBC's	GGA	63.6	[26]	
Fe	Cu	{111}	DFT - PBC's	GGA	54	[22]	
Fe	Mo	Sigma 3 [1-10](111)	DFT - slab	GGA FLAPW	-84.8	[27]	
Fe	P	Sigma 3 [1-10](111)	DFT - slab	GGA – FLAPW	18.3	[27]	
Fe	Mo	Sigma 3 (111)	DFT - slab	GGA		[28]	
Fe	Mo	Sigma 3 {111}	DFT - slab	GGA	-86.7	[28]	
Fe	Mn	Sigma 3 [1-10](111)	DFT - slab	von Barth and Hedin	19.3	[29]	
Fe	Nb	Sigma 3 [1-10](111)	DFT- (Dmol cluster)	GGA	-49.1	[30]	
Fe	Ni	Sigma 5 [001](310)	MD - EAM		-20.2	[25]	
Fe	Pd	Sigma 3 (111)	DFT - slab	GGA		[28]	
Fe	Pd	Sigma 3 {111}	DFT - slab	GGA	7.7	[31]	
Fe	Re	{111}	DFT FLAPW – slab	GGA	-126.2	[24]	
Fe	Re	[001]/(010)	DFT - (Dmol cluster)	GGA	-190.8	[32]	
Fe	Ru	Sigma 3 {111}	DFT FLAPW – slab	GGA	-62.6	[24]	
Fe	Si	{013}	Rice-Wang Estimation	N/A	-9	[33]	
Fe	Sn	Polycrystal Average	Rice-Wang Estimation	N/A	41.5	[5]	
Fe	Ti	[001]/(010)	DFT - Dmol	LDA	-189.8	[34]	
Fe	Ti	[1-10](111)	DFT - DMol	?	-35.8	[35]	

Table 1 (continued): Embrittling potencies for use in quantitative analysis.

Base Metal	Solute	GB	Method	XC Functional, if DFT	ΔE_B(kJ/mol) (Embrittling potency)	Reference	Notes
Fe	Ti	Sigma 11 [1-10]/(11-3)	DFT – LCAO (cluster method)	?	39.5	[36]	In gamma Iron. Study included anyway, as this analysis should be agnostic to crystal structure.
Fe	V	Sigma 3 (111)	DFT – slab	GGA		[28]	
Fe	V	[1-10](111)	DFT- (Dmol cluster)	GGA	-55.9	[30]	
Fe	V	[1-10](111)	DFT – PBC's	GGA	10.6	[37]	Published on arXiv. Included to be thorough. May influence the placement of V on the Fe-V map upward relative to the peer-reviewed data points.
Fe	V	Sigma 5 (210) {111}	MD – EAM	N/A	-25.6	[38]	
Fe	W		DFT FLAPW – slab	GGA	-126.2	[24]	
Fe	Zn	Sigma 3 [001]	DFT – PBC's	GGA – PBE	60.5	[39]	
Fe	Zn	Sigma 5 [001]	DFT – PBC's	GGA – PBE	74.8	[39]	
Fe	B	[1-10](111)	DFT-slab	?		[40]	
Fe	B	Sigma 3 (111)	DFT – slab	GGA	-52.8	[41]	
Fe	B	{111}	DFT – PBC's	GGA (PW91)	-18.3	[42]	
Fe	B	Sigma 5 (210)	DFT – PBC's	GGA (PW91)	-54.9	[42]	
Fe	B	[001]/(010)	DFT – Dmol	LDA	-188.9	[34]	
Fe	B	Sigma 3 (111)	DFT – LMTO			[43]	
Fe	C	Sigma 3 (111)	DFT – slab	GGA	-49.2	[41]	
Fe	C	Sigma 5 [001](310)	MD – EAM		-125.3	[25]	
Fe	C	Sigma 3 (111)	DFT FLAPW	von Barth and Hedin	-58.8	[44]	
Fe	C	Sigma 3 (111)	DFT – PBC's	GGA (PW91)	-7.7	[42]	
Fe	C	{210}	DFT – PBC's	GGA (PW91)	-6.7	[42]	
Fe	C	Polycrystalline average	Rice-Wang Estimation		16.5	[5]	
Fe	C	Sigma 3 (111)	DFT – linear muffin-tin orbitals			[43]	
Fe	P	Sigma 3 (111)	DFT – slab	GGA	27.7	[41]	
Fe	P	Sigma 3 [1-10](111)	DFT – GGA w/ PBE			[45]	
Fe	P	Sigma 3 (111)	DFT – PBC's	GGA	157.1	[22]	

Table 1 (continued): Embrittling potencies for use in quantitative analysis.

Base Metal	Solute	GB	Method	XC Functional, if DFT	ΔE_B(kJ/mol) (Embrittling potency)	Reference	Notes
Fe	P	Sigma 3 (111)	DFT – PBC's	GGA (PW91)	-53	[42]	
Fe	P	Sigma 5 (210)	DFT – PBC's	GGA (PW91)	4.8	[42]	
Fe	P	Sigma 3 [1-10](111)	DFT – slab	von Barth and Hedin	16.4	[29]	
Fe	P	Sigma 5 (210)	MD – EAM	N/A	40.1	[38]	
Fe	P	Sigma 3 [1-10](111)	DFT-slab	?	76.1	[40]	
Fe	P	Sigma 3 [1-10](111)	DFT – slab	GGA	26.8	[46]	
Fe	P	Sigma 3 [1-10](111)	DFT – DVM	LCAO	99.2	[47]	
Fe	P	Polycrystalline average	Rice-Wang Estimation	N/A	41.5	[5]	
Fe	P	Sigma 3 [1-10](111)	DFT – LMTO			[43]	
Fe	P	{013}	Rice-Wang Estimation	N/A	7	[33]	
Fe	Sb	Polycrystal Average	Rice-Wang Estimation		90	[5]	
Fe	Co	Sigma 5 [001](013)	DFT – PBC's	GGA – PBE		[48]	
Fe	Si	Sigma 5 [001](013)	DFT – PBC's	GGA – PBE		[48]	
Fe	V	Sigma 5 [001](013)	DFT – PBC's	GGA – PBE		S[48]	
Fe	Cr	Sigma 5 [001](013)	DFT – PBC's	GGA – PBE		[48]	
Fe	Mn	Sigma 5 [001](013)	DFT – PBC's	GGA – PBE		[48]	
Fe	Ni	Sigma 5 [001](013)	DFT – PBC's	GGA – PBE		[48]	
Fe	Cu	Sigma 5 [001](013)	DFT – PBC's	GGA – PBE		[48]	
Fe	Ti	Sigma 5 [001](013)	DFT – PBC's	GGA – PBE		[48]	
Fe	Mo	Sigma 5 [001](013)	DFT – PBC's	GGA – PBE		[48]	
Fe	Nb	Sigma 5 [001](013)	DFT – PBC's	GGA – PBE		[48]	
Fe	Mn	Sigma 11 [1-10]/(11-3)	DFT w/ LCAO (cluster method)	LCAO	197.5	[36]	In gamma iron. Study included anyway, as this analysis should be agnostic to crystal structure.
Fe	Cr	Sigma 11 [1-10]/(11-3)	DFT w/ LCAO (cluster method)	LCAO	-106	[36]	In gamma iron. Study included anyway, as this analysis should be agnostic to crystal structure.

Table 1 (continued): Embrittling potencies for use in quantitative analysis.

Base Metal	Solute	GB	Method	XC Functional, if DFT	ΔE_B(kJ/mol) (Embrittling potency)	Reference	Notes
Fe	V	Sigma 11 [1-10]/(11-3)	DFT w/ LCAO (cluster method)	LCAO	-59.7	[36]	In gamma iron. Study included anyway, as this analysis should be agnostic to crystal structure.
Fe	N	Sigma 5 (210)	DFT – PBC's	GGA (PW91)	6.7	[42]	
Fe	N	Sigma 3 (111)	DFT – PBC's	GGA (PW91)	9.6	[42]	
Fe	N	[1-10](111)	DFT – DVM	LCAO	-95.4	[47]	
Fe	N	Sigma 3 [1-10](111)	DFT FLAPW-slab	GGA	24.1	[49]	
Fe	O	Sigma 5 (210)	DFT – PBC's	GGA (PW91)	73.2	[42]	
Fe	O	[001]/(010)	DFT – Dmol	LDA	102.1	[34]	
Fe	S	Sigma 3 (111)	DFT – slab	GGA	126.4	[41]	
Fe	S	Sigma 3[1-10](111)	DFT – slab	GGA	133	[46]	
Fe	S	Polycrystalline average	Rice-Wang Estimation		123.5	[5]	
Fe	S	{111}	DFT – linear muffin-tin orbitals			[43]	
Fe	S	{013}	Rice-Wang Estimation		73	[33]	
Fe	S	Sigma 3 (111)[1-10]	DFT – PBC's	GGA (PW91)	35.7	[42]	
Fe	S	Sigma 5 (210)	DFT – PBC's	GGA (PW91)	26	[42]	
Fe	S	Sigma 3 (111)[1-10]	DFT-slab	Not specified (but spin polarized, likely GGA as that is what the authors used previously)	127	[50]	
Fe	S	{111}	DFT – linear muffin-tin orbitals			[43]	
Ni	S	Sigma 5 (210)[100]	DFT-slab	Not specified (but spin polarized, likely GGA as that is what the authors used previously)	138.8	[50]	
Ni	Bi	Sigma 5 (210)[100]	DFT – Slab	GGA – PBE	197.7	[51]	

Table 1 (continued): Embrittling potencies for use in quantitative analysis.

Base Metal	Solute	GB	Method	XC Functional, if DFT	ΔE_B(kJ/mol) (Embrittling potency)	Reference	Notes
Ni	S	Sigma 5 (210)[100]	DFT – Slab	GGA – PBE	118.6	[51]	
Ni	Cr	Sigma 5 (210)[100]	DFT – Slab	GGA – PBE	-23.7	[51]	
Ni	B	Sigma 5 (210)[100]	DFT – Slab	GGA – PBE	-62.5	[51]	
Ni	Y	Sigma 5 (210)[100]	DFT – Slab	GGA – PBE	119.3	[51]	
Ni	Zr	Sigma 5 (210)[100]	DFT – Slab	GGA – PBE	-14	[51]	
Ni	Nb	Sigma 5 (210)[100]	DFT – Slab	GGA – PBE	-74	[51]	
Ni	Mo	Sigma 5 (210)[100]	DFT – Slab	GGA – PBE	-86.3	[51]	
Ni	Tc	Sigma 5 (210)[100]	DFT – Slab	GGA – PBE	-78.5	[51]	
Ni	Ru	Sigma 5 (210)[100]	DFT – Slab	GGA – PBE	-91.3	[51]	
Ni	Rh	Sigma 5 (210)[100]	DFT – Slab	GGA – PBE	-32.7	[51]	
Ni	Pd	Sigma 5 (210)[100]	DFT – Slab	GGA – PBE	8.6	[51]	
Ni	Ag	Sigma 5 (210)[100]	DFT – Slab	GGA – PBE	72.7	[51]	
Ni	La	Sigma 5 (210)[100]	DFT – Slab	GGA – PBE	220.6	[51]	
Ni	Hf	Sigma 5 (210)[100]	DFT – Slab	GGA – PBE	-45.7	[51]	
Ni	Ta	Sigma 5 (210)[100]	DFT – Slab	GGA – PBE	-92.6	[51]	
Ni	W	Sigma 5 (210)[100]	DFT – Slab	GGA – PBE	-116.8	[51]	
Ni	Re	Sigma 5 (210)[100]	DFT – Slab	GGA – PBE	-118.5	[51]	
Ni	Os	Sigma 5 (210)[100]	DFT – Slab	GGA – PBE	-96.7	[51]	
Ni	Ir	Sigma 5 (210)[100]	DFT – Slab	GGA – PBE	-66.6	[51]	
Ni	Pt	Sigma 5 (210)[100]	DFT – Slab	GGA – PBE	-24.8	[51]	
Ni	Au	Sigma 5 (210)[100]	DFT – Slab	GGA – PBE	47.1	[51]	
Ni	Li	Sigma 5 (210)	DFT-Slab FLAPW	GGA	120.4	[52]	
Ni	Ca	Sigma 5 (210)	DFT –Slab FLAPW	GGA	115.6	[52]	
Ni	Ca	Sigma 5 (210)	DFT FLAPW – slab	GGA	159	[24]	
Ni	B	Sigma 5 (210)	DFT FLAPW – slab	GGA	-47.2	[53]	
Ni	P	Sigma 5 (210)	DFT FLAPW – slab	GGA	67.4	[53]	

Table 1 (continued): Embrittling potencies for use in quantitative analysis.

Base Metal	Solute	GB	Method	XC Functional, if DFT	ΔE_B(kJ/mol) (Embrittling potency)	Reference	Notes
Ni	Te	Sigma 5 (210)	DFT-PBC's	GGA	168.6	[54]	
Ni	Nb	Sigma 5 (012)[100]	DFT – PBC's	GGA – PBE	-99.2	[55]	
Ni	Te	Sigma 5 (012)[100]	DFT – PBC's	GGA – PBE	208.1	[55]	
Ni	P	Sigma 5 (012)[100]	DFT – PBC's	GGA		[56]	
Ni	Al	Sigma 5(012)[100]	DFT – PBC's	GGA (spin-polarized)	-3	[57]	
Ni	Si	Sigma 5(012)[100]	DFT – PBC's	GGA (spin-polarized)	-40	[57]	
Ni	P	Sigma 5(012)[100]	DFT – PBC's	GGA (spin-polarized)	-7	[57]	
Ni	S	Sigma 5(012)[100]	DFT – PBC's	GGA (spin-polarized)	96	[57]	
Ni	Ga	Sigma 5(012)[100]	DFT – PBC's	GGA (spin-polarized)	41	[57]	
Ni	Ge	Sigma 5(012)[100]	DFT – PBC's	GGA (spin-polarized)	44	[57]	
Ni	As	Sigma 5(012)[100]	DFT – PBC's	GGA (spin-polarized)	78	[57]	
Ni	Se	Sigma 5(012)[100]	DFT – PBC's	GGA (spin-polarized)	160	[57]	
Ni	In	Sigma 5(012)[100]	DFT – PBC's	GGA (spin-polarized)	49	[57]	
Ni	Sn	Sigma 5(012)[100]	DFT – PBC's	GGA (spin-polarized)	55	[57]	
Ni	Sb	Sigma 5(012)[100]	DFT – PBC's	GGA (spin-polarized)	97	[57]	
Ni	Te	Sigma 5(012)[100]	DFT – PBC's	GGA (spin-polarized)	155	[57]	
Ni	P	Sigma 5(012)[100]	DFT – PBC's	FLAPW (WIEN2k) GGA (spin-polarized)	-12.5	[58]	
Ni	H	Sigma 5(012)[100]	DFT – PBC's	FLAPW (WIEN2k) GGA (spin-polarized)	34	[58]	

Table 1 (continued): Embrittling potencies for use in quantitative analysis.

Base Metal	Solute	GB	Method	XC Functional, if DFT	ΔE_B(kJ/mol) (Embrittling potency)	Reference	Notes
Ni	He	Sigma 5(012)[100]	DFT – PBC's	FLAPW (WIEN2k) GGA (spin-polarized)	265.1	[58]	
Ni	Li	Sigma 5(012)[100]	DFT – PBC's	FLAPW (WIEN2k) GGA (spin-polarized)	84.3	[58]	
Ni	B	Sigma 5(012)[100]	DFT – PBC's	FLAPW (WIEN2k) GGA (spin-polarized)	-207.2	[58]	
Ni	C	Sigma 5(012)[100]	DFT – PBC's	FLAPW (WIEN2k) GGA (spin-polarized)	59.91	[58]	
Ni	N	Sigma 5(012)[100]	DFT – PBC's	FLAPW (WIEN2k) GGA (spin-polarized)	-11.8	[58]	
Ni	Be	Sigma 5(012)[100]	DFT – PBC's	FLAPW (WIEN2k) GGA (spin-polarized)	44.6	[58]	
Ni	O	Sigma 5(012)[100]	DFT – PBC's	FLAPW (WIEN2k) GGA (spin-polarized)	142.3	[58]	
Ni	F	Sigma 5(012)[100]	DFT – PBC's	FLAPW (WIEN2k) GGA (spin-polarized)	203.4	[58]	
Ni	Ne	Sigma 5(012)[100]	DFT – PBC's	FLAPW (WIEN2k) GGA (spin-polarized)	273.6	[58]	
Ni	Na	Sigma 5(012)[100]	DFT – PBC's	FLAPW (WIEN2k) GGA (spin-polarized)	197.3	[58]	
Ni	Mg	Sigma 5(012)[100]	DFT – PBC's	FLAPW (WIEN2k) GGA (spin-polarized)	56.9	[58]	

Table 1 (continued): Embrittling potencies for use in quantitative analysis.

Base Metal	Solute	GB	Method	XC Functional, if DFT	ΔE_B(kJ/mol) (Embrittling potency)	Reference	Notes
Ni	Al	Sigma 5(012)[100]	DFT – PBC's	FLAPW (WIEN2k) GGA (spin-polarized)	-7.2	[58]	
Ni	Si	Sigma 5(012)[100]	DFT – PBC's	FLAPW (WIEN2k) GGA (spin-polarized)	-45.4	[58]	
Ni	P	Sigma 5(012)[100]	DFT – PBC's	FLAPW (WIEN2k) GGA (spin-polarized)	-11.8	[58]	
Ni	S	Sigma 5(012)[100]	DFT – PBC's	FLAPW (WIEN2k) GGA (spin-polarized)	104.2	[58]	
Ni	Cl	Sigma 5(012)[100]	DFT – PBC's	FLAPW (WIEN2k) GGA (spin-polarized)	264.5	[58]	
Ni	Ar	Sigma 5(012)[100]	DFT – PBC's	FLAPW (WIEN2k) GGA (spin-polarized)	407.9	[58]	
Ni	K	Sigma 5(012)[100]	DFT – PBC's	FLAPW (WIEN2k) GGA (spin-polarized)	239.1	[58]	
Ni	Ca	Sigma 5(012)[100]	DFT – PBC's	FLAPW (WIEN2k) GGA (spin-polarized)	140.6	[58]	
Ni	Ga	Sigma 5(012)[100]	DFT – PBC's	FLAPW (WIEN2k) GGA (spin-polarized)	39.1	[58]	
Ni	Ge	Sigma 5(012)[100]	DFT – PBC's	FLAPW (WIEN2k) GGA (spin-polarized)	49.3	[58]	
Ni	As	Sigma 5(012)[100]	DFT – PBC's	FLAPW (WIEN2k) GGA (spin-polarized)	84	[58]	

Table 1 (continued): Embrittling potencies for use in quantitative analysis.

Base Metal	Solute	GB	Method	XC Functional, if DFT	ΔE_B(kJ/mol) (Embrittling potency)	Reference	Notes
Ni	Se	Sigma 5(012)[100]	DFT – PBC's	FLAPW (WIEN2k) GGA (spin-polarized)	173.9	[58]	
Ni	Br	Sigma 5(012)[100]	DFT – PBC's	FLAPW (WIEN2k) GGA (spin-polarized)	255	[58]	
Ni	Kr	Sigma 5(012)[100]	DFT – PBC's	FLAPW (WIEN2k) GGA (spin-polarized)	483.9	[58]	
Ni	Rb	Sigma 5(012)[100]	DFT – PBC's	FLAPW (WIEN2k) GGA (spin-polarized)	233.3	[58]	
Ni	Sr	Sigma 5(012)[100]	DFT – PBC's	FLAPW (WIEN2k) GGA (spin-polarized)	149.2	[58]	
Ni	In	Sigma 5(012)[100]	DFT – PBC's	FLAPW (WIEN2k) GGA (spin-polarized)	52.2	[58]	
Ni	Sn	Sigma 5(012)[100]	DFT – PBC's	FLAPW (WIEN2k) GGA (spin-polarized)	56.5	[58]	
Ni	Sb	Sigma 5(012)[100]	DFT – PBC's	FLAPW (WIEN2k) GGA (spin-polarized)	102	[58]	
Ni	Te	Sigma 5(012)[100]	DFT – PBC's	FLAPW (WIEN2k) GGA (spin-polarized)	169.5	[58]	
Ni	I	Sigma 5(012)[100]	DFT – PBC's	FLAPW (WIEN2k) GGA (spin-polarized)	218.8	[58]	
Ni	Xe	Sigma 5(012)[100]	DFT – PBC's	FLAPW (WIEN2k) GGA (spin-polarized)	337.6	[58]	

Table 1 (continued): Embrittling potencies for use in quantitative analysis.

Base Metal	Solute	GB	Method	XC Functional, if DFT	ΔE_B(kJ/mol) (Embrittling potency)	Reference	Notes
Ni	Cs	Sigma 5(012)[100]	DFT – PBC's	FLAPW (WIEN2k) GGA (spin-polarized)	282.5	[58]	
Ni	Ba	Sigma 5(012)[100]	DFT – PBC's	FLAPW (WIEN2k) GGA (spin-polarized)	176.8	[58]	
Ni	Tl	Sigma 5(012)[100]	DFT – PBC's	FLAPW (WIEN2k) GGA (spin-polarized)	113	[58]	
Ni	Pb	Sigma 5(012)[100]	DFT – PBC's	FLAPW (WIEN2k) GGA (spin-polarized)	120.3	[58]	
Ni	Bi	Sigma 5(012)[100]	DFT – PBC's	FLAPW (WIEN2k) GGA (spin-polarized)	133.3	[58]	
Ni	Po	Sigma 5(012)[100]	DFT – PBC's	FLAPW (WIEN2k) GGA (spin-polarized)	146.3	[58]	
Ni	At	Sigma 5(012)[100]	DFT – PBC's	FLAPW (WIEN2k) GGA (spin-polarized)	176.8	[58]	
Ni	Rn	Sigma 5(012)[100]	DFT – PBC's	FLAPW (WIEN2k) GGA (spin-polarized)	308.6	[58]	
Ni	Sc	Sigma 5(012)[100]	DFT – PBC's	GGA – PBE	54.9	[59]	
Ni	Y	Sigma 5(012)[100]	DFT – PBC's	GGA – PBE	127.2	[59]	
Ni	La	Sigma 5(012)[100]	DFT – PBC's	GGA – PBE	214.4	[59]	
Ni	Ce	Sigma 5(012)[100]	DFT – PBC's	GGA – PBE	210.8	[59]	
Ni	Pr	Sigma 5(012)[100]	DFT – PBC's	GGA – PBE	194.2	[59]	
Ni	Nd	Sigma 5(012)[100]	DFT – PBC's	GGA – PBE	182.9	[59]	

Table 1 (continued): Embrittling potencies for use in quantitative analysis.

Base Metal	Solute	GB	Method	XC Functional, if DFT	ΔE_B(kJ/mol) (Embrittling potency)	Reference	Notes
Ni	Pm	Sigma 5(012)[100]	DFT – PBC's	GGA – PBE	169.9	[59]	
Ni	Sm	Sigma 5(012)[100]	DFT – PBC's	GGA – PBE	160.6	[59]	
Ni	Eu	Sigma 5(012)[100]	DFT – PBC's	GGA – PBE	197.4	[59]	
Ni	Gd	Sigma 5(012)[100]	DFT – PBC's	GGA – PBE	139.3	[59]	
Ni	Tb	Sigma 5(012)[100]	DFT – PBC's	GGA – PBE	131.1	[59]	
Ni	Dy	Sigma 5(012)[100]	DFT – PBC's	GGA – PBE	121.7	[59]	
Ni	Ho	Sigma 5(012)[100]	DFT – PBC's	GGA – PBE	114.5	[59]	
Ni	Er	Sigma 5(012)[100]	DFT – PBC's	GGA – PBE	107.2	[59]	
Ni	Tm	Sigma 5(012)[100]	DFT – PBC's	GGA – PBE	98.9	[59]	
Ni	Yb	Sigma 5(012)[100]	DFT – PBC's	GGA – PBE	162.8	[59]	
Ni	Lu	Sigma 5(012)[100]	DFT – PBC's	GGA – PBE	86.5	[59]	
Ni	He	Sigma 5 [001] Twist Boundary	DFT-PBC's	Not specified (but these authors favor GGA-PBE)	196.6	[60]	Technical Report from Lockheed Martin and Materials Design Inc.
Ni	Li	Sigma 5 [001] Twist Boundary	DFT-PBC's	Not specified (but these authors favor GGA-PBE)	63.6	[60]	Technical Report from Lockheed Martin and Materials Design Inc.
Ni	S	Sigma 5 [001] Twist Boundary	DFT-PBC's	Not specified (but these authors favor GGA-PBE)	50.1	[60]	Technical Report from Lockheed Martin and Materials Design Inc.
Ni	H	Sigma 5 [001] Twist Boundary	DFT-PBC's	Not specified (but these authors favor GGA-PBE)	40.5	[60]	Technical Report from Lockheed Martin and Materials Design Inc.
Ni	C	Sigma 5 [001] Twist Boundary	DFT-PBC's	Not specified (but these authors favor GGA-PBE)	35.7	[60]	Technical Report from Lockheed Martin and Materials Design Inc.

Table 1 (continued): Embrittling potencies for use in quantitative analysis.

Base Metal	Solute	GB	Method	XC Functional, if DFT	ΔE_B(kJ/mol) (Embrittling potency)	Reference	Notes
Ni	Zr	Sigma 5 [001] Twist Boundary	DFT-PBC's	Not specified (but these authors favor GGA-PBE)	7.7	[60]	Technical Report from Lockheed Martin and Materials Design Inc.
Ni	P	Sigma 5 [001] Twist Boundary	DFT-PBC's	Not specified (but these authors favor GGA-PBE)	-9.6	[60]	Technical Report from Lockheed Martin and Materials Design Inc.
Ni	Fe	Sigma 5 [001] Twist Boundary	DFT-PBC's	Not specified (but these authors favor GGA-PBE)	-16.3	[60]	Technical Report from Lockheed Martin and Materials Design Inc.
Ni	Mn	Sigma 5 [001] Twist Boundary	DFT-PBC's	Not specified (but these authors favor GGA-PBE)	-19.3	[60]	Technical Report from Lockheed Martin and Materials Design Inc.
Ni	Nb	Sigma 5 [001] Twist Boundary	DFT-PBC's	Not specified (but these authors favor GGA-PBE)	-44.3	[60]	Technical Report from Lockheed Martin and Materials Design Inc.
Ni	Cr	Sigma 5 [001] Twist Boundary	DFT-PBC's	Not specified (but these authors favor GGA-PBE)	-51.1	[60]	Technical Report from Lockheed Martin and Materials Design Inc.
Ni	B	Sigma 5 [001] Twist Boundary	DFT-PBC's	Not specified (but these authors favor GGA-PBE)	-99.2	[60]	Technical Report from Lockheed Martin and Materials Design Inc.
Mg	Ag	Near Sigma 7 (12-30[001] 21.8 deg STGB	DFT - PBC's	GGA - PBE		[61]	
Mg	Zn	Near Sigma 7 (12-30[001] 21.8 deg STGB	DFT - PBC's	GGA - PBE		[61]	
Mg	Al	Near Sigma 7 (12-30[001] 21.8 deg STGB	DFT – PBC's	GGA – PBE		[61]	
Mg	Cd	Near Sigma 7 (12-30[001] 21.8 deg STGB	DFT – PBC's	GGA – PBE		[61]	
Mg	Y	Near Sigma 7 (12-30[001] 21.8 deg STGB	DFT – PBC's	GGA – PBE		[61]	

Table 1 (continued): Embrittling potencies for use in quantitative analysis.

Base Metal	Solute	GB	Method	XC Functional, if DFT	ΔE_B(kJ/mol) (Embrittling potency)	Reference	Notes
Mg	Ca	Near Sigma 7 (12-30[001] 21.8 deg STGB	DFT – PBC's	GGA – PBE		[61]	
Mg	Nd	Near Sigma 7 (12-30[001] 21.8 deg STGB	DFT – PBC's	GGA – PBE		[61]	
Mg	Ce	Near Sigma 7 (12-30[001] 21.8 deg STGB	DFT – PBC's	GGA – PBE		[61]	
Mg	La	Near Sigma 7 (12-30[001] 21.8 deg STGB	DFT – PBC's	GGA – PBE		[61]	
Mg	Ti	Near Sigma 7 (12-30[001] 21.8 deg STGB	DFT – PBC's	GGA – PBE		[61]	
Mg	Zr	Near Sigma 7 (12-30[001] 21.8 deg STGB	DFT – PBC's	GGA – PBE		[61]	
Mg	Ag	{10-12} tension twin	DFT – PBC's	GGA-PBE		[62]	
Mg	Al	{10-12} tension twin	DFT – PBC's	GGA-PBE		[62]	
Mg	Ce	{10-12} tension twin	DFT – PBC's	GGA-PBE		[62]	
Mg	Dy	{10-12} tension twin	DFT – PBC's	GGA-PBE		[62]	
Mg	Er	{10-12} tension twin	DFT – PBC's	GGA-PBE		[62]	
Mg	Ga	{10-12} tension twin	DFT – PBC's	GGA-PBE		[62]	
Mg	Gd	{10-12} tension twin	DFT – PBC's	GGA-PBE		[62]	
Mg	Ho	{10-12} tension twin	DFT – PBC's	GGA-PBE		[62]	
Mg	Li	{10-12} tension twin	DFT – PBC's	GGA-PBE		[62]	
Mg	Lu	{10-12} tension twin	DFT – PBC's	GGA-PBE		[62]	
Mg	Mn	{10-12} tension twin	DFT – PBC's	GGA-PBE		[62]	
Mg	Nd	{10-12} tension twin	DFT – PBC's	GGA-PBE		[62]	
Mg	Pb	{10-12} tension twin	DFT – PBC's	GGA-PBE		[62]	
Mg	Sc	{10-12} tension twin	DFT – PBC's	GGA-PBE		[62]	
Mg	Sm	{10-12} tension twin	DFT – PBC's	GGA-PBE		[62]	
Mg	Sn	{10-12} tension twin	DFT – PBC's	GGA-PBE		[62]	

Table 1 (continued): Embrittling potencies for use in quantitative analysis.

Base Metal	Solute	GB	Method	XC Functional, if DFT	ΔE_B(kJ/mol) (Embrittling potency)	Reference	Notes
Mg	Tl	{10-12} tension twin	DFT – PBC’s	GGA-PBE		[62]	
Mg	Y	{10-12} tension twin	DFT – PBC’s	GGA-PBE		[62]	
Mg	Yb	{10-12} tension twin	DFT – PBC’s	GGA-PBE		[62]	
Mg	Zn	{10-12} tension twin	DFT – PBC’s	GGA-PBE		[62]	
Mg	Zr	{10-12} tension twin	DFT – PBC’s	GGA-PBE		[62]	
Mg	Na	Sigma 7 [0001](12-30)	DFT-PBC’s	FLAPW – Unknown	22.9	[63]	
Mg	Y	Sigma 7 [0001](12-30)	DFT-PBC’s	FLAPW – Unknown	-80.5	[63]	
Mg	Al	Sigma 7 [0001](12-30)	DFT-PBC’s	FLAPW – Unknown	-22.5	[63]	
Mg	Zn	Sigma 7 [0001](12-30)	DFT-PBC’s	FLAPW – Unknown	-19.5	[63]	
Mg	Li	Sigma 7 [0001](12-30)	DFT-PBC’s	FLAPW – Unknown	12.3	[63]	
Mg	Zr	Sigma 7 [0001](12-30)	DFT-PBC’s	FLAPW – Unknown		[63]	
Mo	B	Sigma 5 (310)[001]	DFT –PBC’s	Mixed local and plane wave basis. LDA?	-104.1	[64]	
Mo	N	Sigma 5 (310)[001]	DFT –PBC’s	Mixed local and plane wave basis. LDA?	78	[64]	
Mo	O	Sigma 5 (310)[001]	DFT –PBC’s	Mixed local and plane wave basis. LDA?	140.7	[64]	
Mo	C	Sigma 5 (310)[001]	DFT (Local) –PBC’s	Mixed local and plane wave basis. LDA?	-29.9	[64]	
Mo	C	Sigma 5 (310)[001]	DFT – PBC’s	GGA	0.9	[65]	
Mo	Ti	Not Specified	DFT	Not specified	2.9	[66]	Private communication from Questek Innovations, LLC.
Mo	Zr	Not Specified	DFT	Not specified	25.1	[66]	Private communication from Questek Innovations, LLC.

Table 1 (continued): Embrittling potencies for use in quantitative analysis.

Base Metal	Solute	GB	Method	XC Functional, if DFT	ΔE_B(kJ/mol) (Embrittling potency)	Reference	Notes
Mo	V	Not Specified	DFT	Not specified	-15.4	[66]	Private communication from Questek Innovations, LLC.
Mo	Nb	Not Specified	DFT	Not specified	-1	[66]	Private communication from Questek Innovations, LLC.
Ti	C	Sigma 7 [0001](12-30)	DFT - PBC's	GGA		[67]	
Ti	N	Sigma 7 [0001](12-30)	DFT - PBC's	GGA		[67]	
Ti	O	Sigma 7 [0001](12-30)	DFT - PBC's	GGA		[67]	
W	Zr	Sigma 27 {525} STGB	DFT-slab	GGA	-70.6	[68]	
W	Nb	Sigma 27 {525} STGB	DFT-slab	GGA	-43.3	[68]	
W	Mo	Sigma 27 {525} STGB	DFT-slab	GGA	-0.8	[68]	
W	Ru	Sigma 27 {525} STGB	DFT-slab	GGA	-31.8	[68]	
W	Rh	Sigma 27 {525} STGB	DFT-slab	GGA	-9.1	[68]	
W	Pd	Sigma 27 {525} STGB	DFT-slab	GGA	35.5	[68]	
W	Hf	Sigma 27 {525} STGB	DFT-slab	GGA	-80.5	[68]	
W	Ta	Sigma 27 {525} STGB	DFT-slab	GGA	-48.3	[68]	
W	Re	Sigma 27 {525} STGB	DFT-slab	GGA	-51.8	[68]	
W	Os	Sigma 27 {525} STGB	DFT-slab	GGA	-55.9	[68]	
W	Ir	Sigma 27 {525} STGB	DFT-slab	GGA	-44.3	[68]	
W	Pt	Sigma 27 {525} STGB	DFT-slab	GGA	-10.5	[68]	
W	Be	Sigma 3 {112}	DFT - PBC's	GGA	271.7	[69]	
W	Be	Sigma 27 {525} STGB	DFT - PBC's	GGA	-49.8	[69]	
W	Li	Sigma 3 {112}	DFT - PBC's	GGA	541.5	[69]	
W	Li	Sigma 27 {525} STGB	DFT - PBC's	GGA	136.6	[69]	
W	H	Sigma 3 {112}	DFT - PBC's	GGA	66.4	[69]	
W	H	Sigma 27 {525} STGB	DFT - PBC's	GGA	58.5	[69]	
W	He	Sigma 3 {112}	DFT - PBC's	GGA	495	[69]	
W	He	Sigma 27 {525} STGB	DFT - PBC's	GGA	272	[69]	
W	Re	Sigma 3 [110](112)	DFT-slab	GGA - PBEsol	-1.8	[70]	
W	Re	Sigma 3 [110](111)	DFT-slab	GGA - PBEsol	-20.7	[70]	

Table 1 (continued): Embrittling potencies for use in quantitative analysis.

Base Metal	Solute	GB	Method	XC Functional, if DFT	ΔE_B(kJ/mol) (Embrittling potency)	Reference	Notes
W	Th	Sigma 3 (111)	DFT-slab	GGA	233.1	[71]	
W	Sr	Sigma 3 (111)	DFT-slab	GGA	464	[71]	
W	Au	Sigma 3 (111)	DFT-slab	GGA	112.1	[71]	
W	In	Sigma 3 (111)	DFT-slab	GGA	180	[71]	
W	Cd	Sigma 3 (111)	DFT-slab	GGA	173.7	[71]	
W	Mn	Sigma 3 (111)	DFT-slab	GGA	-57.8	[71]	
W	Cu	Sigma 3 (111)	DFT-slab	GGA	-21.3	[71]	
W	Ag	Sigma 3 (111)	DFT-slab	GGA	143.4	[71]	
W	Sc	Sigma 3 (111)	DFT-slab	GGA	123.6	[71]	
W	Zn	Sigma 3 (111)	DFT-slab	GGA	17.3	[71]	
W	Cr	Sigma 3 (111)	DFT-slab	GGA	-34.9	[71]	
W	Ti	Sigma 3 (111)	DFT-slab	GGA	54.8	[71]	
Zr	Cs	Sigma 7 (0001)/(0001) twist boundary (36°)	DFT - Slab	GGA - PBE	468.9	[72]	
Zr	I	Sigma 7 (0001)/(0001) twist boundary (36°)	DFT - Slab	GGA - PBE	250.3	[72]	
Zr	He	Sigma 7 (0001)/(0001) twist boundary (36°)	DFT - Slab	GGA - PBE	217.6	[72]	
Zr	Te	Sigma 7 (0001)/(0001) twist boundary (36°)	DFT - Slab	GGA - PBE	123.6	[72]	
Zr	Sb	Sigma 7 (0001)/(0001) twist boundary (36°)	DFT - Slab	GGA - PBE	116.4	[72]	
Zr	Li	Sigma 7 (0001)/(0001) twist boundary (36°)	DFT - Slab	GGA - PBE	103.2	[72]	
Zr	O	Sigma 7 (0001)/(0001) twist boundary (36 degrees)	DFT - Slab	GGA - PBE	68.1	[72]	
Zr	Sn	Sigma 7 (0001)/(0001) twist boundary (36 degrees)	DFT - Slab	GGA - PBE	69.8	[72]	
Zr	Cd	Sigma 7 (0001)/(0001) twist boundary (36 degrees)	DFT - Slab	GGA - PBE	56.3	[72]	
Zr	H	Sigma 7 (0001)/(0001) twist boundary (36 degrees)	DFT - Slab	GGA - PBE	46	[72]	
Zr	Si	Sigma 7 (0001)/(0001) twist boundary (36 degrees)	DFT - Slab	GGA - PBE	30.4	[72]	

Table 1 (continued): Embrittling potencies for use in quantitative analysis.

Base Metal	Solute	GB	Method	XC Functional, if DFT	ΔE_B(kJ/mol) (Embrittling potency)	Reference	Notes
Zr	C	Sigma 7 (0001)/(0001) twist boundary (36 degrees)	DFT - Slab	GGA - PBE	20.7	[72]	
Zr	N	Sigma 7 (0001)/(0001) twist boundary (36 degrees)	DFT - Slab	GGA - PBE	14.7	[72]	
Zr	B	Sigma 7 (0001)/(0001) twist boundary (36 degrees)	DFT - Slab	GGA - PBE	4.4	[72]	
Zr	U	Sigma 7 (0001)/(0001) twist boundary (36 degrees)	DFT - Slab	GGA - PBE	7.3	[72]	
Zr	Ni	Sigma 7 (0001)/(0001) twist boundary (36 degrees)	DFT - Slab	GGA - PBE	4.9	[72]	
Zr	Hf	Sigma 7 (0001)/(0001) twist boundary (36 degrees)	DFT - Slab	GGA - PBE	-0.3	[72]	
Zr	Nb	Sigma 7 (0001)/(0001) twist boundary (36 degrees)	DFT - Slab	GGA - PBE	-10.2	[72]	
Zr	Cr	Sigma 7 (0001)/(0001) twist boundary (36 degrees)	DFT - Slab	GGA - PBE	-16.5	[72]	
Zr	Fe	Sigma 7 (0001)/(0001) twist boundary (36 degrees)	DFT - Slab	GGA - PBE	-33.1	[72]	

In similar spirit to the inclusion of studies in Table 1 where an embrittling potency was not calculated, Table 2 lists calculations and their associated references that are deemed not as suitable for quantitative analysis. These are nonetheless listed so that the interested reader may more easily find studies on grain boundary segregation and embrittlement for specific systems.

Table 2: Embrittling potencies possibly not suitable for quantitative analysis.

Base Metal	Solute	GB	Method	XC Functional, if DFT	ΔE_B(kJ/mol)	Reference	Notes
Ni	V	Sigma 5 (012)[100]	DFT - Slab	Norm-Conserving Pseudopotentials with LDA	72.3	[73]	Methodology unclear.
Fe	Cr	Sigma 5 (210)	DFT - PBC'S	GGA	-14.9	[74]	Methodology unclear

Table 2 (continued): Calculations not included in the quantitative analysis.

Base Metal	Solute	GB	Method	XC Functional, if DFT	ΔE_{B}(kJ/mol)	Reference	Notes
Fe	Mo	Sigma 5 (210)	DFT - PBC'S	GGA	10.1	[74]	Methodology unclear
Fe	Nb	Sigma 5 (210)	DFT - PBC'S	GGA	19.5	[74]	Methodology unclear
Fe	Mn	Sigma 11 [1-10]/(11-3)	DFT DMOL	LCAO	197.5	[75]	Periodic calculations preferred to cluster calculations.
Fe	Cr	Sigma 11 [1-10]/(11-3)	DFT DMOL	LCAO	-106	[75]	Periodic calculations preferred to cluster calculations.
Fe	N	N/A	DFT with LDA - bad for Fe			[76]	Use of results from LDA for Fe is not preferred, as it predicts the wrong ground state. As there are results for Fe-N using GGA, this result was excluded.
Fe	P	Sigma 3 [1-10](111)	DFT DMOL		95.4	[77]	Many results on Fe-P are available which use periodic boundary conditions, which are preferred.
Ni	H	Sigma 5 (210)	DFT FLAPW - slab	GGA	0.3	[53]	Results in W show that choice of interstitial site is crucial for H, so this result was excluded, as a thorough site analysis was not available.
Fe	H	Sigma 5 [001](310)	MD - EAM	N/A	144.5	[25]	It is unclear the extent to which the physics of H segregation can be captured by EAM, so this result was excluded.
Ni	He	Sigma 5 (210)	DFT-Slab FLAPW	GGA	240.9	[52]	Site occupation not clear, and there are calculations which examined multiple sites for He in Ni, so this result was excluded.

Table 2 (continued): Calculations not included in the quantitative analysis.

Base Metal	Solute	GB	Method	XC Functional, if DFT	ΔE_B(kJ/mol)	Reference	Notes
Fe	N	{111}	MD - Finnis-Sinclair		139	[78]	Excluded based on the recommendation of Ref [6].
Fe	O	{111}	MD - Finnis-Sinclair		132	[78]	Excluded based on the recommendation of Ref [6].
Fe	S	{111}	MD - Finnis-Sinclair		556	[78]	Excluded based on the recommendation of Ref [6].
Fe	H	{111}	MD - Finnis-Sinclair		23	[78]	Excluded based on the recommendation of Ref [6].
Fe	P	{111}	MD - Finnis-Sinclair		355	[78]	Excluded based on the recommendation of Ref [6].
Fe	C	{111}	MD - Finnis-Sinclair		68	[78]	Excluded based on the recommendation of Ref [6].
Fe	B	{111}	MD - Finnis-Sinclair		63	[78]	Excluded based on the recommendation of Ref [6].
Fe	Si	{111}	MD - Finnis-Sinclair		244	[78]	Excluded based on the recommendation of Ref [6].
Fe	O	Sigma 5 (210)	DFT - PBC's	GGA (PW91)	72.3	[79]	These authors later came out with a more thorough study
Fe	N	Sigma 5 (210)	DFT - PBC's	GGA (PW91)	-19.3	[79]	These authors later came out with a more thorough study
Cu	He	Sigma 5 (310)[001]	DFT			Unpublished. Mentioned in [20]	Said to be embrittling.
Cu	Kr	Sigma 5 (310)[001]	DFT			Unpublished. Mentioned in [20]	Said to be embrittling.
Al	Co	3 twist and 3 tilt GB's	MD - EAM	N/A	N/A	[80]	Calculations could not be interpreted in terms of embrittling potencies.

Table 2 (continued): Calculations not included in the quantitative analysis.

Base Metal	Solute	GB	Method	XC Functional, if DFT	ΔE_B(kJ/mol)	Reference	Notes
Al	Fe	4 twist and 3 tilt GB's	MD - EAM	N/A	N/A	[80]	Calculations could not be interpreted in terms of embrittling potencies.
Al	Ti	5 twist and 3 tilt GB's	MD - EAM	N/A	N/A	[80]	Calculations could not be interpreted in terms of embrittling potencies.
Al	Mg	6 twist and 3 tilt GB's	MD - EAM	N/A	N/A	[80]	Calculations could not be interpreted in terms of embrittling potencies.
Al	Cu	7 twist and 3 tilt GB's	MD - EAM	N/A	N/A	[80]	Calculations could not be interpreted in terms of embrittling potencies.
Al	Pb	8 twist and 3 tilt GB's	MD - EAM	N/A	N/A	[80]	Calculations could not be interpreted in terms of embrittling potencies.
Ni	W	Sigma 5 (210)	DFT-slab	GGA	-127.2	[81]	Repeats many of the calculations in Ref [51]
Ni	Zr	Sigma 5 (210)	DFT-slab	GGA	-16.4	[81]	Repeats many of the calculations in Ref [51]
Ni	Hf	Sigma 5 (210)	DFT-slab	GGA	-47.2	[81]	Repeats many of the calculations in Ref [51]
Ni	Bi	Sigma 5 (210)	DFT-slab	GGA	194.6	[81]	Repeats many of the calculations in Ref [51]
Ni	S	Sigma 5 (210)	DFT-slab	GGA	105	[81]	Repeats many of the calculations in Ref [51]
Ni	B	Sigma 5 (210)	DFT-slab	GGA	-67.4	[81]	Repeats many of the calculations in Ref [51]
Ni	Ta	Sigma 5 (210)	DFT-slab	GGA	-102.1	[81]	Repeats many of the calculations in Ref [51]
Ni	Re	Sigma 5 (210)	DFT-slab	GGA	-128.2	[81]	Repeats many of the calculations in Ref [51]

Table 2 (continued): Calculations not included in the quantitative analysis.

Base Metal	Solute	GB	Method	XC Functional, if DFT	ΔE_B(kJ/mol)	Reference	Notes
W	H	Sigma 3 (111)	DFT-not specified	Not specified	25.8	[82]	Methodology unclear
W	B	Sigma 3 (111)	DFT-not specified	Not specified	-17.6	[82]	Methodology unclear
W	C	Sigma 3 (111)	DFT-not specified	Not specified	-55.2	[82]	Methodology unclear
W	N	Sigma 3 (111)	DFT-not specified	Not specified	36.4	[82]	Methodology unclear
W	O	Sigma 3 (111)	DFT-not specified	Not specified	150.4	[82]	Methodology unclear
W	F	Sigma 3 (111)	DFT-not specified	Not specified	341.9	[82]	Methodology unclear
W	Al	Sigma 3 (111)	DFT-not specified	Not specified	285.5	[82]	Methodology unclear
W	Si	Sigma 3 (111)	DFT-not specified	Not specified	245.6	[82]	Methodology unclear
W	P	Sigma 3 (111)	DFT-not specified	Not specified	255	[82]	Methodology unclear
W	S	Sigma 3 (111)	DFT-not specified	Not specified	358.4	[82]	Methodology unclear
W	Cl	Sigma 3 (111)	DFT-not specified	Not specified	575.8	[82]	Methodology unclear
Ta	H	Sigma 3 (111)	DFT-not specified	Not specified	56.4	[82]	Methodology unclear
Ta	B	Sigma 3 (111)	DFT-not specified	Not specified	-17.6	[82]	Methodology unclear
Ta	C	Sigma 3 (111)	DFT-not specified	Not specified	-84.7	[82]	Methodology unclear
Ta	N	Sigma 3 (111)	DFT-not specified	Not specified	-22.3	[82]	Methodology unclear
Ta	O	Sigma 3 (111)	DFT-not specified	Not specified	47	[82]	Methodology unclear
Ta	F	Sigma 3 (111)	DFT-not specified	Not specified	275	[82]	Methodology unclear
Ta	Al	Sigma 3 (111)	DFT-not specified	Not specified	179.8	[82]	Methodology unclear

Table 2 (continued): Embrittling potencies not included in the quantitative analysis.

Base Metal	Solute	GB	Method	XC Functional, if DFT	ΔE_B(kJ/mol)	Reference	Notes
Ta	Si	Sigma 3 (111)	DFT- not specified	Not specified	108.1	[82]	Methodology unclear
Ta	P	Sigma 3 (111)	DFT- not specified	Not specified	110.5	[82]	Methodology unclear
Ta	S	Sigma 3 (111)	DFT- not specified	Not specified	162.1	[82]	Methodology unclear
Ta	Cl	Sigma 3 (111)	DFT- not specified	Not specified	372.5	[82]	Methodology unclear
Fe	B	Sigma 5 (210)	DFT - PBC's	GGA (PW91)	-55.9	[79]	This is the value for a substitutional site. The same authors later showed that the substitutional and interstitial sites exhibit opposite signs for the change in cohesion at the GB, and that the interstitial site is preferred.

References in Tables 1 and 2

7. Lu, G.H., et al., *Origin of intergranular embrittlement of Al alloys induced by Na and Ca segregation: Grain boundary weakening.* Physical Review B, 2006. **73**(22): p. 5.
8. Uesugi, T. and K. Higashi, *Segregation of Alkali and Alkaline Earth Metals at Sigma 11(113) 110 Grain Boundary in Aluminum from First-Principles Calculations.* Materials Transactions, 2012. **53**(9): p. 1699-1705.
9. Razumovskiy, V.I., et al., *The effect of alloying elements on grain boundary and bulk cohesion in aluminum alloys: An ab initio study.* Scripta Materialia, 2011. **65**(10): p. 926-929.
10. Zhang, S.J., et al., *Cohesion enhancing effect of magnesium in aluminum grain boundary: A first-principles determination.* Applied Physics Letters, 2012. **100**(23): p. 4.
11. Liu, X.G., et al., *First-principles investigation of Mg segregation at Sigma=11(113) grain boundaries in Al.* Journal of Physics-Condensed Matter, 2005. **17**(27): p. 4301-4308.
12. Zhang, S.J., et al., *Sodium-induced embrittlement of an aluminum grain boundary.* Physical Review B, 2010. **82**(22): p. 17.
13. Zhang, S.J., et al., *Aluminum grain boundary decohesion by dense sodium segregation.* Physical Review B, 2012. **85**(21): p. 8.
14. Zhang, S.J., et al., *First-principles determination of the effect of boron on aluminum grain boundary cohesion.* Physical Review B, 2011. **84**(13): p. 9.
15. Zhang, S.J., et al., *First principles investigation of zinc-induced embrittlement in an aluminum grain boundary.* Acta Materialia, 2011. **59**(15): p. 6155-6167.

16. Ossowski, T., E. Wachowicz, and A. Kiejna, *Effect of iron additions on intergranular cohesion in chromium.* Journal of Physics-Condensed Matter, 2009. **21**(48): p. 8.
17. Lozovoi, A.Y., A.T. Paxton, and M.W. Finnis, *Structural and chemical embrittlement of grain boundaries by impurities: A general theory and first-principles calculations for copper.* Physical Review B, 2006. **74**(15): p. 13.
18. Duscher, G., et al., *Bismuth-induced embrittlement of copper grain boundaries.* Nature Materials, 2004. **3**(9): p. 621-626.
19. Schweinfest, R., A.T. Paxton, and M.W. Finnis, *Bismuth embrittlement of copper is an atomic size effect.* Nature, 2004. **432**(7020): p. 1008-1011.
20. Lozovoi, A.Y. and A.T. Paxton, *Boron in copper: A perfect misfit in the bulk and cohesion enhancer at a grain boundary.* Physical Review B, 2008. **77**(16): p. 14.
21. Wimmer, A., et al., *Temperature dependent transition of intragranular plastic to intergranular brittle failure in electrodeposited Cu micro-tensile samples.* Materials Science and Engineering a-Structural Materials Properties Microstructure and Processing, 2014. **618**: p. 398-405.
22. Yuasa, M. and M. Mabuchi, *First-principles study in Fe grain boundary with Al segregation: variation in electronic structures with straining.* Philosophical Magazine, 2013. **93**(6): p. 635-647.
23. Shang, J.X., et al., *Effects of Co and Cr on bcc Fe grain boundaries cohesion from first-principles study.* Computational Materials Science, 2006. **38**(1): p. 217-222.
24. Geng, W.T., A.J. Freeman, and G.B. Olson, *Influence of alloying additions on grain boundary cohesion of transition metals: First-principles determination and its phenomenological extension.* Physical Review B, 2001. **63**(16): p. 9.
25. Farkas, D., et al., *Atomistic simulations of the effects of segregated elements on grain-boundary fracture in body-centered-cubic Fe.* Metallurgical and Materials Transactions a-Physical Metallurgy and Materials Science, 2005. **36A**(8): p. 2067-2072.
26. Yuasa, M. and M. Mabuchi, *Effects of segregated Cu on an Fe grain boundary by first-principles tensile tests.* Journal of Physics-Condensed Matter, 2010. **22**(50): p. 7.
27. Geng, W.T., A.J. Freeman, and G.B. Olson, *Influence of alloying additions on the impurity induced grain boundary embrittlement.* Solid State Communications, 2001. **119**(10-11): p. 585-590.
28. Tian, Z.X., et al., *Effect of alloying additions on the hydrogen-induced grain boundary embrittlement in iron.* Journal of Physics-Condensed Matter, 2011. **23**(1): p. 8.
29. Zhong, L.P., et al., *Effects of Mn additions on the P embrittlement of the Fe grain boundary.* Physical Review B, 1997. **55**(17): p. 11133-11137.
30. Shang, J.X. and C.Y. Wang, *Electronic effects of alloying elements Nb and V on body-centred-cubic Fe grain boundary cohesion.* Journal of Physics-Condensed Matter, 2001. **13**(42): p. 9635-9644.
31. Geng, W.T., et al., *Effect of Mo and Pd on the grain-boundary cohesion of Fe.* Physical Review B, 2000. **62**(10): p. 6208-6214.
32. Chen, Z.Z. and C.Y. Wang, *Effect of element Re on the grain boundary cohesion of alpha-Fe.* Chinese Physics, 2006. **15**(3): p. 604-609.
33. Lejcek, P., S. Hofmann, and A. Krajnikov, *Chemical aspects of brittle fracture: grain boundary segregation.* Materials Science and Engineering a-Structural Materials Properties Microstructure and Processing, 1997. **234**: p. 283-286.
34. Chen, Z.Z. and C.Y. Wang, *First-principles study on the effects of co-segregation of Ti, B and O on the cohesion of the alpha-Fe grain boundary.* Journal of Physics-Condensed Matter, 2005. **17**(42): p. 6645-6652.
35. Shang, J.X., D.L. Zhao, and C.Y. Wang, *Effect of titanium on bcc Fe grain boundary cohesion.* Acta Metallurgica Sinica, 2001. **37**(8): p. 893-896.

36. Yang, R., et al., *The effects of 3d alloying elements on grain boundary cohesion in gamma-iron: a first principles study on interface embrittlement due to the segregation.* Journal of Physics-Condensed Matter, 2003. **15**(49): p. 8339-8349.
37. Kim, S., et al., *The effects of vanadium on the strength of a bcc Fe Σ3(111)[1-10] grain boundary*, 2012: arXiv.
38. Rajagopalan, M., M.A. Tschopp, and K.N. Solanki, *Grain Boundary Segregation of Interstitial and Substitutional Impurity Atoms in Alpha-Iron.* Jom, 2014. **66**(1): p. 129-138.
39. Bauer, K.D., et al., *A first principles investigation of zinc induced embrittlement at grain boundaries in bcc iron.* Acta Materialia, 2015. **90**: p. 69-76.
40. Wu, R.Q., A.J. Freeman, and G.B. Olson, *First principles determination of the effects of phosporus and boron on iron grain-boundary cohesion.* Science, 1994. **265**(5170): p. 376-380.
41. Yamaguchi, M., *First-Principles Study on the Grain Boundary Embrittlement of Metals by Solute Segregation: Part I. Iron (Fe)-Solute (B, C, P, and S) Systems.* Metallurgical and Materials Transactions a-Physical Metallurgy and Materials Science, 2011. **42A**(2): p. 319-329.
42. Wachowicz, E. and A. Kiejna, *Effect of impurities on structural, cohesive and magnetic properties of grain boundaries in alpha-Fe.* Modelling and Simulation in Materials Science and Engineering, 2011. **19**(2): p. 20.
43. Krasko, G.L. and G.B. Olson, *Effect of boron, carbon, phosphorus, and sulfur on intergranular cohesion in iron.* Solid State Communications, 1990. **76**(3): p. 247-251.
44. Wu, R.Q., A.J. Freeman, and G.B. Olson, *Effects of carbon on Fe-grain-boundary cohesion: First-principles determination.* Physical Review B, 1996. **53**(11): p. 7504-7509.
45. Yuasa, M. and M. Mabuchi, *Bond mobility mechanism in grain boundary embrittlement: First-principles tensile tests of Fe with a P-segregated Sigma 3 grain boundary.* Physical Review B, 2010. **82**(9): p. 5.
46. Yamaguchi, M., Y. Nishiyama, and H. Kaburaki, *Decohesion of iron grain boundaries by sulfur or phosphorous segregation: First-principles calculations.* Physical Review B, 2007. **76**(3): p. 5.
47. Fen, Y.Q. and C.Y. Wang, *Electronic effects of nitrogen and phosphorus on iron grain boundary cohesion.* Computational Materials Science, 2001. **20**(1): p. 48-56.
48. Jin, H., I. Elfimov, and M. Militzer, *Study of the interaction of solutes with Sigma 5 (013) tilt grain boundaries in iron using density-functional theory.* Journal of Applied Physics, 2014. **115**(9): p. 8.
49. Kim, M., C.B. Geller, and A.J. Freeman, *The effect of interstitial N on grain boundary cohesive strength in Fe.* Scripta Materialia, 2004. **50**(10): p. 1341-1343.
50. Yamaguchi, M., M. Shiga, and H. Kaburaki, *Grain boundary decohesion by sulfur segregation in ferromagnetic iron and nickel - A first-principles study.* Materials Transactions, 2006. **47**(11): p. 2682-2689.
51. Razumovskiy, V.I., A.Y. Lozovoi, and I.M. Razurnovskii, *First-principles-aided design of a new Ni-base superalloy: Influence of transition metal alloying elements on grain boundary and bulk cohesion.* Acta Materialia, 2015. **82**: p. 369-377.
52. Smith, R.W., et al., *The effect of Li, He and Ca on grain boundary cohesive strength in Ni.* Scripta Materialia, 2000. **43**(10): p. 957-961.
53. Geng, W.T., et al., *Embrittling and strengthening effects of hydrogen, boron, and phosphorus on a Sigma 5 nickel grain boundary.* Physical Review B, 1999. **60**(10): p. 7149-7155.
54. Liu, W.G., et al., *First-principles study of intergranular embrittlement induced by Te in the Ni Sigma 5 grain boundary.* Computational Materials Science, 2014. **88**: p. 22-27.
55. Liu, W.G., et al., *The effect of Nb additive on Te-induced stress corrosion cracking in Ni alloy: a first-principles calculation.* Nuclear Science and Techniques, 2014. **25**(5): p. 5.
56. Liu, W.G., et al., *First-principles study of the effect of phosphorus on nickel grain boundary.* Journal of Applied Physics, 2014. **115**(4): p. 7.

57. Vsianska, M. and M. Sob, *The effect of segregated sp-impurities on grain-boundary and surface structure, magnetism and embrittlement in nickel.* Progress in Materials Science, 2011. **56**(6): p. 817-840.
58. Yamaguchi, M., M. Shiga, and H. Kaburaki, *Energetics of segregation and embrittling potency for non-transition elements in the Ni Sigma 5 (012) symmetrical tilt grain boundary: a first-principles study.* Journal of Physics-Condensed Matter, 2004. **16**(23): p. 3933-3956.
59. Liu, W.G., et al., *Effects of rare-earth on the cohesion of Ni Sigma 5 (012) grain boundary from first-principles calculations.* Computational Materials Science, 2015. **96**: p. 374-378.
60. Young, G.A., et al., *An Atomistic Modeling Study of Alloying Element, Impurity Element, and Transmutation Products on the Cohesion of a Nickel Σ5 {001} Twist Grain Boundary*, 2003, Lockhhed Martin Corporation and Materials Design Inc.
61. Huber, L., J. Rottler, and M. Militzer, *Atomistic simulations of the interaction of alloying elements with grain boundaries in Mg.* Acta Materialia, 2014. **80**: p. 194-204.
62. Zhang, J., Y.C. Dou, and Y. Zheng, *Twin-boundary segregation energies and solute-diffusion activation enthalpies in Mg-based binary systems: A first-principles study.* Scripta Materialia, 2014. **80**: p. 17-20.
63. Olson, G.B. and S. Zhang, *Ductilization of High-Strength Magnesium Alloys*, 2012, US Military.
64. Janisch, R. and C. Elsasser, *Segregated light elements at grain boundaries in niobium and molybdenum.* Physical Review B, 2003. **67**(22): p. 11.
65. Tahir, A.M., R. Janisch, and A. Hartmaier, *Ab initio calculation of traction separation laws for a grain boundary in molybdenum with segregated C impurites.* Modelling and Simulation in Materials Science and Engineering, 2013. **21**(7): p. 16.
66. Sebastian, J., M.A. Gibson, Editor 2015.
67. Aksyonov, D.A., A.G. Lipnitskii, and Y.R. Kolobov, *Grain boundary segregation of C, N and O in hexagonal close-packed titanium from first principles.* Modelling and Simulation in Materials Science and Engineering, 2013. **21**(7): p. 12.
68. Setyawan, W. and R.J. Kurtz, *Effects of transition metals on the grain boundary cohesion in tungsten.* Scripta Materialia, 2012. **66**(8): p. 558-561.
69. Setyawan, W. and R.J. Kurtz, *Ab initio study of H, He, Li and Be impurity effect in tungsten Sigma 3{112} and Sigma 27{552} grain boundaries.* Journal of Physics-Condensed Matter, 2014. **26**(13).
70. Scheiber, D., et al., *Ab initio description of segregation and cohesion of grain boundaries in W-25 at.% Re alloys.* Acta Materialia, 2015. **88**: p. 180-189.
71. Li, Z.W., et al., *Segregation of alloying atoms at a tilt symmetric grain boundary in tungsten and their strengthening and embrittling effects.* Chinese Physics B, 2014. **23**(10): p. 6.
72. Christensen, M., et al., *Effect of impurity and alloying elements on Zr grain boundary strength from first-principles computations.* Journal of Nuclear Materials, 2010. **404**(2): p. 121-127.
73. Bentria, E., I. Lefkaier, and B. Bentria, *The effect of vanadium impurity on Nickel Sigma 5(012) grain boundary.* Materials Science and Engineering a-Structural Materials Properties Microstructure and Processing, 2013. **577**: p. 197-201.
74. Li, C.X., et al., *Effect of Cr, Mo, and Nb additions on intergranular cohesion of ferritic stainless steel: First-principles determination.* Chinese Physics B, 2014. **23**(3): p. 6.
75. Yang, R., et al., *Effects of Cr, Mn on the cohesion of the gamma-iron grain boundary.* Acta Materialia, 2001. **49**(6): p. 1079-1085.
76. Braithwaite, J.S. and P. Rez, *Grain boundary impurities in iron.* Acta Materialia, 2005. **53**(9): p. 2715-2726.
77. Sagert, L.P., G.B. Olson, and D.E. Ellis, *Chemical embrittlement of Fe grain boundaries: P and the P-Mo couple.* Philosophical Magazine B-Physics of Condensed Matter Statistical Mechanics Electronic Optical and Magnetic Properties, 1998. **77**(3): p. 871-889.

78. Krasko, G.L. *Energetics of ideal grain boundary fracture in iron and the thermodynamic criterion of impurity embrittlement*. in *Symposium on Interfacial Engineering for Optimized Properties*. 1996. Boston, Ma: Materials Research Society.
79. Wachowicz, E. and A. Kiejna, *Effect of impurities on grain boundary cohesion in bcc iron.* Computational Materials Science, 2008. **43**(4): p. 736-743.
80. Babicheva, R.I., et al., *Effect of grain boundary segregations of Fe, Co, Cu, Ti, Mg and Pb on small plastic deformation of nanocrystalline Al.* Computational Materials Science, 2015. **98**: p. 410-416.
81. Razumovskiy, V.I., et al., *Analysis of the alloying system in Ni-base superalloys based on ab initio study of impurity segregation to Ni grain boundary.* Euro Superalloys 2010, 2011. **278**: p. 192-197.
82. Pan, Z.L., L.J. Kecskes, and Q.M. Wei, *The nature behind the preferentially embrittling effect of impurities on the ductility of tungsten.* Computational Materials Science, 2014. **93**: p. 104-111.
